# The hidden price of repeated traumatic exposure: different cognitive deficits in different first-responders

**DOI:** 10.3389/fnbeh.2014.00281

**Published:** 2014-08-20

**Authors:** Einat Levy-Gigi, Gal Richter-Levin, Szabolcs Kéri

**Affiliations:** ^1^The institute for the Study of Affective Neuroscience, University of HaifaHaifa, Israel; ^2^Nyírö Gyula Hospital, National Psychiatry and Addiction CenterBudapest, Hungary; ^3^Department of Psychology, University of HaifaHaifa, Israel; ^4^Sagol Department of Neurobiology, University of HaifaHaifa, Israel; ^5^Department of Physiology, Faculty of Medicine, University of SzegedSzeged, Hungary; ^6^Department of Cognitive Science, Budapest University of Technology and EconomicsBudapest, Hungary

**Keywords:** reversal-learning, context, cue, repeated traumatic exposure, hippocampus, first-responders, firefighters, CSI police

## Abstract

Studies on first responders who are repeatedly exposed to traumatic events report low levels of PTSD symptoms and diagnosis. However, neuroimaging and behavioral studies show that traumatic exposure is associated with brain and cognitive dysfunctions. Taking together it may suggest that traumatic exposure have a price, which is not sufficiently defined by the standard PTSD measures. In a recent study we revealed that similar to individuals with PTSD, non-PTSD highly exposed firefighters display a selective impairment in hippocampal related functions. In the current study we aimed to test whether different first responders display a similar impairment. We concentrated on unique populations of active duty firefighters and criminal scene-investigators (CSI) police, who are frequently exposed to similar levels and types of traumatic events, and compared them to civilian matched-controls with no history of trauma-exposure. We used a hippocampal dependent cue-context reversal paradigm, which separately evaluates reversal of negative and positive outcomes of cue and context related information. We predicted and found that all participants were equally able to acquire and retain stimulus-outcome associations. However, there were significant differences in reversal learning between the groups. Performance among firefighters replicated our prior findings; they struggled to learn that a previously negative context is later associated with a positive outcome. CSI police on the other hand showed a selective impairment in reversing the outcome of a negative cue. Hence after learning that a specific cue is associated with a negative outcome, they could not learn that later it is associated with a positive outcome. Performance in both groups did not correlate with levels of PTSD, anxiety, depression or behavioral inhibition symptoms. The results provide further evidence of the hidden price of traumatic exposure, suggesting that this price may differ as a function of occupation.

## Introduction

A conventional view of the possible effects of traumatic stress classifies trauma- exposed individuals into two major groups: those who suffer from post-traumatic stress disorder (PTSD) and those who remain resilient. Interestingly, studies on first responders who are repeatedly exposed to traumatic events as part of their daily routine, report low levels of PTSD symptoms in this group (e.g., Pole et al., 2009; Soo et al., 2011; Fushimi, 2012; Meyer et al., 2012; Orr et al., 2012; Admon et al., 2013). These findings may lead to the conclusion that highly exposed individuals are especially resilient to the possible aversive effects of repeated traumatic exposure. However, this conclusion does not take into account that repeated traumatic exposure might have a hidden price. Specifically, it may affect different behavioral, cognitive and brain related functions and structures that are not sufficiently defined by the standard measures of PTSD symptoms and diagnosis. The aim of the present study is to provide further evidence for the hidden price of traumatic exposure by testing highly trauma exposed non-PTSD first responders from different occupations.

The probable price of traumatic exposure is reflected in both animal and human studies, which show a significant decrease in brain related structure and function after exposure to potential traumatic events (e.g., Woodward et al., 2009, 2013; Clinchy et al., 2011; Papagni et al., 2011; Chen et al., 2012; Goswami et al., 2012; Yuen et al., 2012; Sekiguchi et al., 2013; for review see Lupien et al., 2009; Fa et al., 2014). While these brain related deficits do not necessarily correlate with levels of PTSD symptoms, recent studies have shown associations between it and the amount of traumatic exposure. Specifically, it was found that greater traumatic exposure is associated with vaster levels of structural and functional changes in the brain (Papagni et al., 2011; Aupperle et al., 2013; Nooner et al., 2013). These results may suggest a significant effect of traumatic exposure, which is not sufficiently defined by the standard measures of PTSD symptoms.

Many of the studies on non-PTSD trauma exposed individuals revealed deficits in hippocampal function and structure. It was shown that not only individuals with PTSD but also trauma-exposed individuals without PTSD have a reduced hippocampal volume compared to trauma-unexposed controls (e.g., Winter and Irle, 2004; for meta-analysis see Karl et al., 2006; Woon et al., 2010). Attempts to explain the mechanisms behind the deficits in hippocampal structure and function in trauma exposed individuals relay mostly on evidence from animal models (Rudy, 2009; Goosens, 2011; Moustafa et al., 2013 for review see Acheson et al., 2012; Maren et al., 2013). According to these models hippocampal deficits may lead to impaired learning and use of associations between contextual information and aversive events. These deficits may explain, for example, why a person who was exposed to a terror attack (e.g., explosion) in a coffee shop may associate all coffee shops and every load sound with a negative outcome.

In order to further test possible relationship between trauma exposure and hippocampal related deficits we developed a novel cue-context reversal paradigm (Levy-Gigi et al., 2014). In a common reversal paradigm, participants acquire a stimulus-outcome association (S→Positive) and later need to reverse the outcome of the same stimulus (S→Negative). However, this paradigm does not take into account that every stimulus occurs in a specific context (Mayes et al., 1992). In our paradigm, participants learn cue + context-outcome associations (A hat on an orange background→Positive) and later view new associations, which require reversing the outcome of *either* the *cue* (A phone on an orange background→Negative) or the *context* (A hat on a gray background→Negative) of the acquired stimuli. This unique innovative manipulation enables us to detect selective impairments in reversing positive and negative outcomes of cue and context related information.

In previous studies we showed that performance on our paradigm significantly correlates with hippocampal functions (Levy-Gigi et al., 2011) and volume reduction (Levy-Gigi et al., 2014). Applying this paradigm in trauma exposed individuals we showed that similar to fully diagnosed PTSD individuals, non-PTSD trauma-exposed firefighters displayed a selective deficit in reversing the outcome of negative context. After they learned that a specific context is associated with a negative outcome, they struggled to learn that the same context predicts a positive outcome when presented later with a new cue (Levy-Gigi and Richter-Levin, 2014; Levy-Gigi et al., 2014). Interestingly, however, we found no significant associations between performance and levels of PTSD symptoms. These results further support a possible hidden price of repeated traumatic exposure in non- PTSD individuals, which may reflect deficits in hippocampal related structure and function.

This possible hidden price of traumatic exposure was demonstrated so far only among firefighters. It can be claimed that since firefighters are trained to focus and react to aversive environmental conditions, they are more aware of the context and ignore other elements. Therefore, they display impaired reversal in negative context, but not cue, conditions. The aim of the present study is to test whether the impaired reversal of negative context is unique to firefighters, or does it also characterize other populations of first responders. To that end, we concentrated on two unique populations of active-duty firefighters and criminal scene investigators (CSI) police. Interestingly, although these two groups play different roles, they are frequently exposed to similar traumatic events. For example- both firefighters and CSI police can be called in cases of fires and car accidents, firefighters would have to extinguish fire and rescue trapped people, while CSI police would need to look for evidence in order to identify the responsible for the fire/accident. To provide a baseline performance we compared these two groups to trauma-unexposed controls, matched for gender, age and education. Participants were tested on our novel cue-context reversal paradigm (Levy-Gigi and Richter-Levin, 2014; Levy-Gigi et al., 2014). Based on previous findings we postulate that both groups would equally learn and retain positive and negative stimulus-outcome associations. However, similar to our prior results, we expect that both firefighters and CSI police would show a selective impairment in reversing the outcome of negative context compared to trauma-unexposed controls.

## Methods and materials

### Participants

35 trauma-exposed firefighters, 32 trauma-exposed CSI police and 23 unexposed matched healthy controls matched for gender, age and years of education volunteered to participate in the study (see Table 1 for a detailed description of the sample).

**Table 1:** **Demographic characteristics of trauma exposed firefighters, CSI police and trauma-unexposed matched controls**.

	**Firefighters (*N* = 35)**	**CSI-Police (*N* = 32)**	**Controls (*N* = 23)**
Age (years)	36.94 (8.63)	39.89 (7.4)	37.26 (6.25)
Male/female	32/3	28/4	19/4
Education (years)	12.4 (.85)	13.06 (1.52)	12.57 (1.12)
Single/married/divorced	7/18/10	4/23/5	3/19/1
Medications^*^	2/35	1/32	1/23

* *Supplementary medications such as benzodiazepines*.

Firefighters and CSI police were randomly recruited from different fire stations and police units, which are all located in a similar setting within a radius of 40 miles. All firefighters and CSI police reported multiple exposure to potential traumatic events as defined in DSM-V Criterion A. Participants in the unexposed control group were civilians who work in an industrial factory. They were recruited by a clinical psychologist who interviewed them to ensure no past exposure to DSM-V criteria A events. Participants in all groups showed high rates of consent (~95%). All participants were interviewed using the Structured Clinical Interview for *Diagnostic and Statistical Manual for Mental Disorders-Forth Edition* (*DSM–IV*) Axis I Disorders (SCID-CV) (First et al., 1996). Exclusion criteria included any current DSM-IV psychopathology including PTSD, and any history of psychiatric or neurological disorders, alcohol abuse or dependence. Three firefighters and one CSI police were excluded from the sample due to a clear diagnosis of PTSD. The Non-PTSD firefighters and CSI police were also interviewed using the Clinician Administrated PTSD Scale (CAPS) (Blake et al., 1995) to assess the levels of PTSD symptoms. All interviews were conducted by a well-trained and regularly supervised clinical psychologist. Finally, based on the Deployment Risk and Resilience Inventory (Vogt et al., 2013) we used a modified list of 8 potential traumatic events (e.g., fires, car accidents, exposure to dead bodies and bodies parts) to assess type and frequency of traumatic exposure. Firefighters and CSI police had to rank their level of exposure on a 1–6 Likert scale (1-never; 6-every day or almost every day).

As can be seen in Table 2 there were no differences between the two groups in average number of years in service, levels of traumatic exposure and levels of PTSD symptoms. The study was carried out in accordance with the Declaration of Helsinki and was approved by the local ethics board. All participants provided a written informed consent at the beginning of the experiment.

**Table 2:** **Trauma exposure and PTSD symptoms (mean and standard deviation) of firefighters and CSI police**.

	**Firefighters (*N* = 35)**	**CSI-Police (*N* = 32)**
Number of years in service	10.23 (9.92)	8.84 (6.17)
Traumatic exposure (Range 8–48)	39.46 (0.61)	39.38 (0.78)
CAPS score (Range 0–136)	18.71 (19.75)	13.44 (12.66)

### Tools

#### Cue and context reversal paradigm

**Figure 1:**
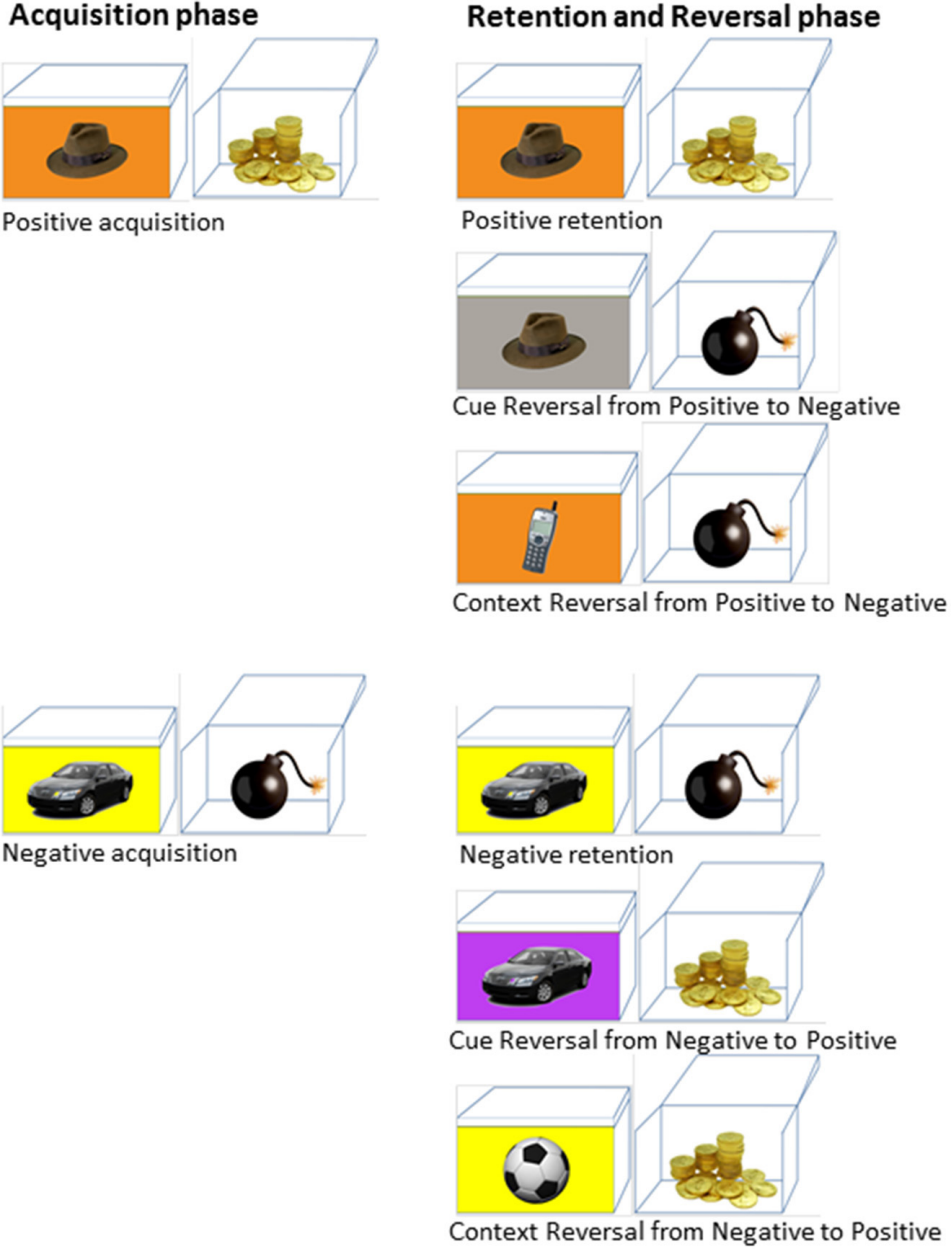
**Example of the stimuli in the two phases of the paradigm**.

**Figure 2:**
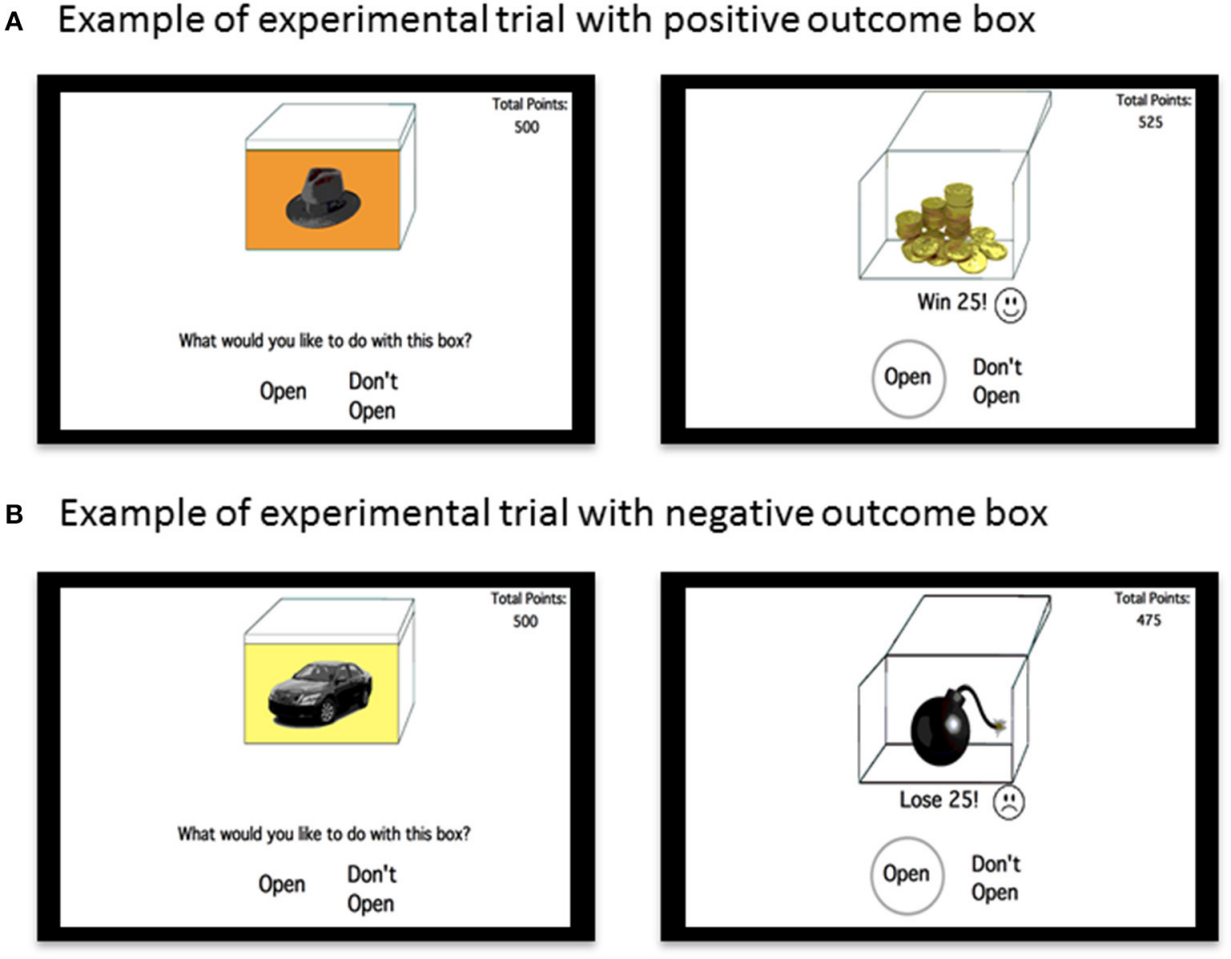
**Example of experimental trials in which participants chose to (A) open a positive-outcome box and (B) open a negative-outcome box**.

A detailed description of the paradigm can be found elsewhere (Levy-Gigi et al., 2014). In this paradigm participants view a series of boxes on a computer screen (Figure 1). On each box there is a picture of a target cue (one of various objects, e.g., a *hat*) presented against a background context (different colors, e.g., *orange*). When opened, each box is associated with a positive or negative outcome (Figure 2). The paradigm has two phases (See Table 3 for a schematic summary of the paradigm). In the acquisition phase, participants learn by trial and error to predict the outcome of four different boxes (i.e., open the two positive boxes and skip the two negative boxes). Each box has a unique cue and context (i.e., a box with a *hat* on an *orange* background has *gold* inside while a box with a *car* on a *yellow* background has *bomb* inside). In order to complete the acquisition phase and move on to the next retention and reversal phase, participants need to learn the box-outcome associations to a criterion of 6 consecutive correct responses. Correct responses refer to conditions in which participants open positive boxes or leave negative boxes closed. Similarly, incorrect responses refer to conditions in which participants open negative boxes or leave positive boxes closed. A subsequent retention and reversal phase starts immediately after the acquisition phase without any signaled switch or delay. In this phase participants receive retention trials with the original boxes that keep the same learned outcome (e.g., a *hat* on an *orange* background has *gold* inside) in addition to two new types of boxes that share either the cue (e.g., a *hat* on a gray background) or the context (e.g., a phone on an *orange* background) with an original box (Figure 1). The new boxes are associated with the opposite outcome relative to the original boxes (i.e., if the box with the *hat* on the *orange* background has *gold* inside, then the boxes with the *hat* on a *gray* background and a *phone* on the *orange* background will have *bomb* inside and vice versa). Therefore, in order to successfully learn these new associations, participants need to reverse the association rule of either the original cue or the original context. Boxes in this phase are presented in 10 blocks of 12 boxes each (two boxes from each of the following conditions: positive/negative retention, positive/negative cue reversal, positive/negative context reversal). This sums up to a total of 120 trials; 20 trials per condition. At the end of the task participants see their total earned points.

**Table 3:**
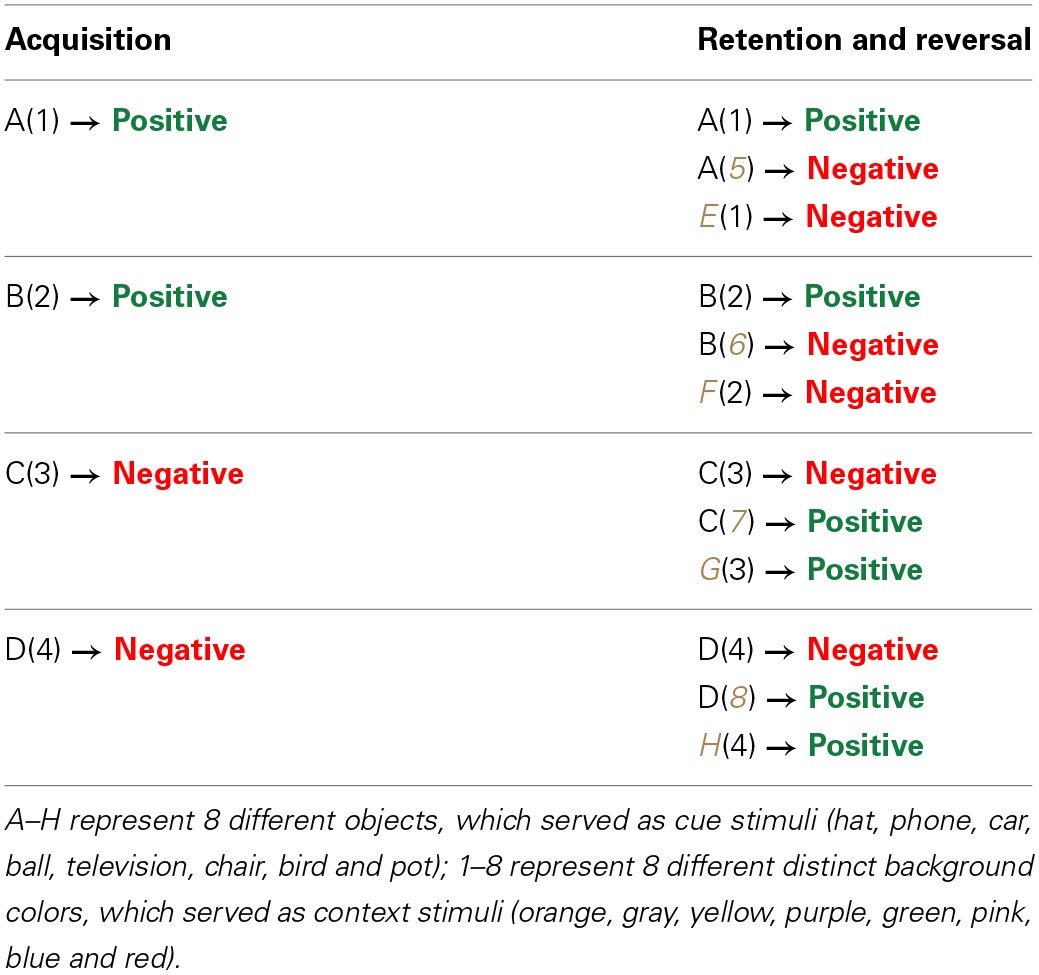
**A schematic summary of the paradigm**.

*A–H represent 8 different objects, which served as cue stimuli (hat, phone, car, ball, television, chair, bird and pot); 1–8 represent 8 different distinct background colors, which served as context stimuli (orange, gray, yellow, purple, green, pink, blue and red)*.

#### Self-report questionnaires and cognitive assessment

All participants completed the following self-report questionnaires in order to control for possible confounds: the revised version of the Beck Depression Inventory (BDI–II; Beck et al., 1996) was used to assess depressive symptoms over the previous 2 weeks. The STAI (State–Trait Anxiety Inventory; Spielberger et al., 1983) was used to assess current and general anxiety. The Hope questionnaire (Snyder et al., 1991), the Adult/Retrospective Measure of Behavioral Inhibition (AMBI/RMBI; Gladstone and Parker, 2005) and the Toronto Alyxethemia Scale (TAS-20; Bagby et al., 1994) were used in order to test whether hope, avoidance and alexithymia correlate with either PTSD symptoms or reversal learning. In addition, we used the scaled scores of the Wechsler Adult Intelligence Scale III (WAIS-III) Blocks Design subtest to estimate IQ levels (Wechsler, 1997). Previous studies showed that scores from this subtest are the best predictor of full IQ scale scores (Spreen, 1998).

### Data analysis

We used SPSS (version 19) software (SPSS Inc., Chicago, IL) to analyze the data. All data were checked for normality of distribution using Kolmogorov–Smirnov tests. Since participants are instructed to open boxes when they first see it, in our analyses we did not include the first response to each new box in the acquisition and reversal trials (note that retention trials include only old boxes, and therefore all trials are analyzed). This was done in order to avoid artificial errors (i.e., when participants open a negative box for the first time) and possible effects of task compliancy.

## Results

### Acquisition and retention of stimulus–outcome associations

**Figure 3:**
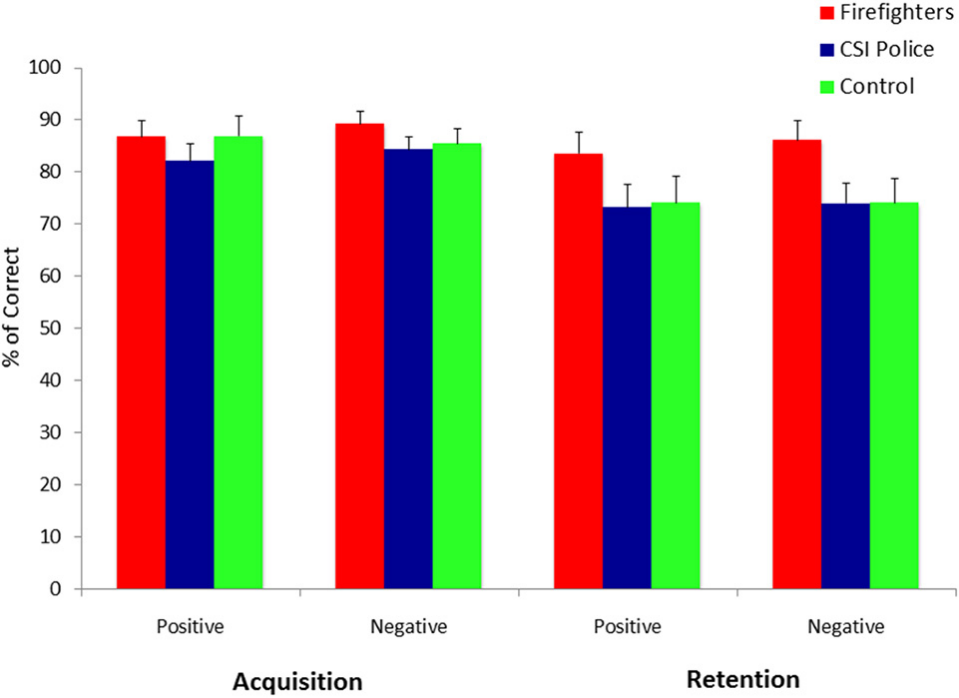
**Percentage of correct responses to the four original boxes as a function of Phase (acquisition vs. retention), Outcome (positive vs. negative) and Experimental Group (trauma exposed firefighters vs. trauma exposed CSI police vs. trauma-unexposed controls)**.

We conducted a group (firefighters, CSI police and unexposed controls) by Acquisition (positive vs. negative stimuli) by Retention (positive vs. negative stimuli) mixed model ANOVA on the percentage of correct responses. In this model, Group was the between-subjects factor, while Acquisition and Retention were the within-subjects factors. The results are depicted in Figure 3. As predicted the ANOVA revealed no significant interactions of Acquisition by Group [*F*_(2, 87)_ = 2.76, *p* > 0.05] Retention by Group [*F*_(2, 87)_ = 0.2, *p* > 0.05], nor Acquisition by Retention By Group [*F*_(2, 87)_ = 0.05, *p* > 0.05]. These results indicate that all groups were equally able to learn and retain positive and negative stimulus-outcome associations.

### Cue and context reversal

**Figure 4:**
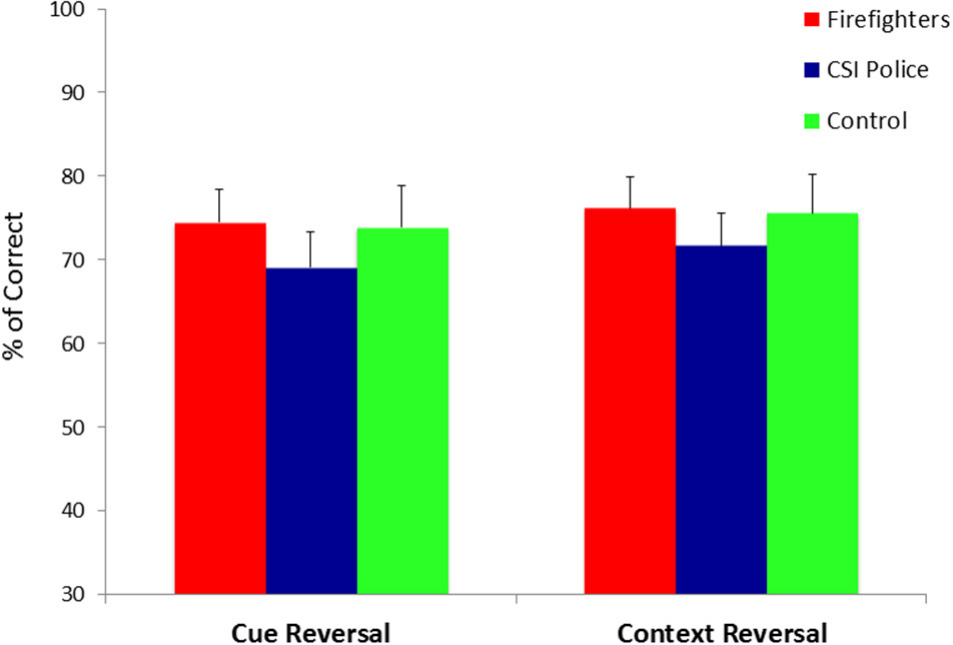
**Percentage of correct responses in the reversal phase as a function of Reversal Type (cue vs. context), and experimental group (trauma exposed firefighters, trauma exposed CSI police and trauma-unexposed controls) in positive to negative trials**.

**Figure 5:**
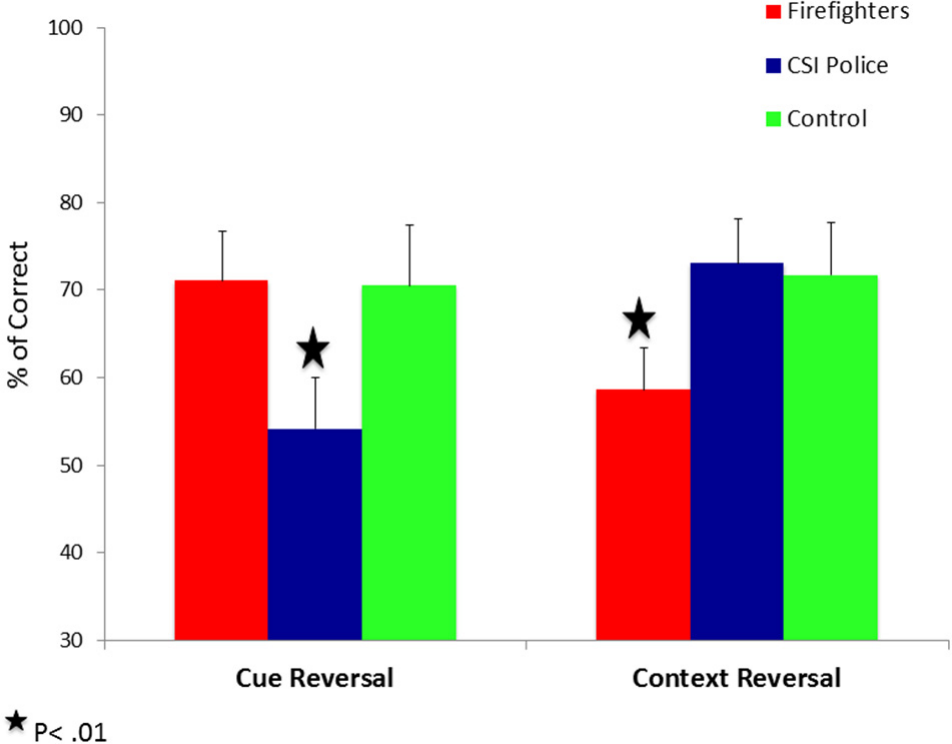
**Percentage of correct responses in the reversal phase as a function of Reversal Type (cue vs. context), and Experimental Group (trauma exposed firefighters, trauma exposed CSI police and trauma-unexposed controls) in negative to positive reversal trials**.

We conducted a Group (firefighters, CSI police and unexposed controls) by Outcome (positive vs. negative feedback) by Reversal Type (cue vs. context) mixed model ANOVA on the percentage of correct responses. In this model Group was the between-subjects factor while Outcome and Reversal Type were the within-subjects factor. The ANOVA revealed a significant triple interaction between Group, Outcome and Reversal Type [*F*_(2, 87)_ = 3.25, *p* < 0.05, η^2^_*p*_ = 0.07]. Follow-up ANOVAs revealed that in positive to negative reversal conditions the interaction between Group and Reversal Type was not significant [*F*_(2, 87)_ = 0.14, *p* = 0.87]. These results indicate that participants from all groups were equally able to learn that cue/context with positive outcome are associated with negative outcome when presented later as part of a new combination (Figure 4). However, in the negative to positive reversal conditions there was a significant interaction between Group and Reversal Type [*F*_(2, 87)_ = 4.77, *p* < 0.05, η^2^_*p*_ = 0.10] (Figure 5). Pairwise comparisons with Bonferroni correction (α = 0.01) showed that CSI police display a selective impairment in negative to positive cue reversal [*F*_(1, 31)_ = 4.65, *p* < 0.05, η^2^_*p*_ = 0.13]; while firefighters display a selective impairment in negative to positive context reversal [*F*_(1, 34)_ =5.5, *p* < 0.05, η^2^_*p*_ = 0.14]. Unexposed matched controls preform equally well in both conditions [*F*_(1, 22)_ = 0.2, *p* = 0.89].

### Self-report questionnaires and cognitive assessment

Table 4 depicts the comparison between firefighters, CSI police and unexposed matched controls on the self- report questionnaires and cognitive assessment. We conducted One-Way ANOVA followed by Scheffe *post-hoc* comparisons to test differences between the groups. There were no significant differences between firefighters and CSI police on all measures, indicating that in many ways these two populations of first responders are very similar. As can be seen there were no differences between the three groups in estimated levels of IQ. In addition, all three groups displayed similar levels of state and trait anxiety symptoms (see also Myers et al., 2013). Moreover, both firefighters and CSI police displayed lower levels of depression compared to unexposed controls. These results, together with their tendency toward external thinking and stronger difficulty to describe their feelings, may suggest that active duty first responders are more focused on their duties and missions, and less involved in introspective thinking. In addition, it is possible that their professional role adds to a positive self- perception that protects them and helps them keep low levels of anxiety and depression (Langan-Fox and Cooper, 2011). However, it should be noted that both groups of first responders had lower levels of hope, suggesting that they are less determined in achieving their goals and the ways in which they can achieve them (Snyder et al., 1991). Finally, similar to other studies (e.g., Myers et al., 2013) we found no differences between the groups in retroactive measure of behavioral inhibition, while both firefighters and CSI police demonstrated higher levels of adulthood behavioral inhibition compared to matched-controls. These results suggest that repeated traumatic exposure may increase the tendency of active duty first responders to avoid unfamiliar places and people, upsurge their feeling of isolation and keep them more alert and cautious. Most importantly, akin with our previous findings (Levy-Gigi and Richter-Levin, 2014) none of the self-report measures correlated with performance on any of the cue-context reversal paradigm conditions.

**Table 4:** **Self-report questionnaires and cognitive assessment (Means and Standard Deviation) of trauma-exposed firefighters, CSI police and trauma unexposed matched controls**.

	**Firefighters**	**CSI Police**	**Controls**
Depression	3.6 (4.29)	2.31 (3)	7.87 (7.08)^*^
State anxiety	27.66 (8.02)	27.72 (9.2)	27 (6.5)
Trait anxiety	26.94 (8.15)	28.44 (6.44)	25.74 (5.33)
Hope	28.37 (2.72)	27.91 (2.97)	30.3 (1.61)^*^
AMBI	11.37 (4.68)	12.16 (4.06)	7.78 (2.8)^*^
RMBI	8.09 (3.5)	9.82 (2.82)	8.26 (2.52)
TAS-difficulty to describe feelings	15.57 (5.35)	13.34 (4.23)	8.65 (3.74)^*^
TAS-difficulty to identify feelings	7.94 (2.9)	7.52 (2.2)	6.43 (3.51)
TAS-external thinking	17.57 (3.43)	17.35 (3.92)	12.35 (3.56)^*^
Estimated IQ	11.43 (2.02)	10.97 (2.38)	11.91 (3.03)

* *Indicates significant differences between means at the p < 0.05 based on Scheffe's post-hoc paired comparisons*.

## Discussion

The aim of the present study was to test the hidden price of repeated traumatic exposure among first responders from different occupations. To that end, we compared the performance of non-PTSD trauma- exposed firefighters and CSI police, and trauma- unexposed matched controls on a novel hippocampal- dependent cue-context reversal paradigm. As predicted, we found that all groups were equally able to learn and retain positive and negative stimulus-outcome associations. In addition, we replicated our previous findings (Levy-Gigi and Richter-Levin, 2014), showing that firefighters have a selective deficit in reversing the outcome of negative context. Specifically, after they learned that a specific context is associated with a negative outcome (e.g., a car on a *yellow* background is *negative*) they could not learn that it predicts positive outcome when presented later with a new cue (e.g., a football on a *yellow* background is *positive*). However, contrary to our predictions, CSI police showed an opposite pattern. Their ability to reverse the outcome of previously negative context was intact; hence, their performance was equal to the performance of unexposed control participants. Nevertheless, they showed a selective impairment in reversing the outcome of a negative cue. Specifically, they struggled to learn that a cue, which was first associated with a negative outcome (e.g., a *car* on a yellow background is *negative)*, is associated with a positive outcome when presented later in a different context (e.g., a *car* on a purple background is *positive*).

The results of the present study provide further support for the associations between repeated traumatic exposure and impaired reversal learning in non-PTSD individuals (Levy-Gigi and Richter-Levin, 2014). The results suggest that similar to individuals with PTSD (Levy-Gigi and Kéri, 2012; Levy-Gigi et al., 2012, 2014; Brown et al., 2013) individuals with repeated traumatic exposure fail to appropriately encode traumatic associations in its adequate context. The fact that this impairment was independent of PTSD symptoms and diagnosis adds to the existing evidence regarding a possible hidden price of repeated traumatic exposure, which is not reflected in the standard measures of PTSD. Moreover, since we used neutral and not trauma related stimuli, it suggests that the price of repeated traumatic exposure is not limited to trauma related conditions. Instead, it reflects a more general impairment, which may affect the way first responders interpret and react to various aspects in their environment.

Contrary to our prediction, the results show that the specific type of impairment varies as a function of occupation. Although these differences between firefighters and CSI police are aligned with animal models that show heterogeneity in response to stressful events (Duvarci et al., 2009; Galatzer-Levy et al., 2013), it is still questionable whether it reflects different aspect of the same brain mechanism or distinctive brain mechanisms. It can be claimed that since the performance in the two trauma-exposed groups reflect impaired learning and use of associations between contextual information and aversive events, it is related to deficit in hippocampal function and structure. Hence, impairments in reversing the outcome of a negative context may explain for example, why a person who was exposed to a terror attack in a coffee shop, may associate all coffee shops with a negative outcome. On the other hand, impairments in reversing the outcome of a negative cue may explain for example why a person who fought in a battle field when there was a loud noise (e.g., explosion) may associate every loud noise (e.g., fireworks) with a negative outcome even if it takes place in a different, safe environment.

Alternatively, it can be claimed that these impairments relate to distinct brain mechanisms. Hence, while impaired context reversal learning may reflect a reduction in hippocampal function and structure (for meta analyses see Kitayama et al., 2005; Smith, 2005; Karl et al., 2006; Woon et al., 2010), impaired cue reversal learning may reflect dysfunction of the amygdala (Saddoris et al., 2005; Schoenbaum and Roesch, 2005). This explanation is in line with findings in animals, showing that lesions to the amygdala result in impaired cue conditioning (Phillips and LeDoux, 1992; LaBar and LeDoux, 1996; Ito et al., 2006). Finally, it is possible that these impairments reflect deficits in Hippocampus-Amygdala connectivity, which differs as a function of occupation. Future fMRI study in non-PTSD trauma exposed firefighters and CSI police is needed in order to determine between these alternative explanations.

The discrepancies between firefighters and police may also result from the unique nature of these two occupations. Although firefighters and CSI police are exposed to similar traumatic events and participants from both groups reported similar levels of traumatic exposure, there are several important disparities in their job requirements. First, they usually arrive to the scenes at different times. While firefighters attend to the emergency short time after it happened, CSI police often attend it later, usually after the danger has passed. Second, these two groups play different roles and need to complete distinct tasks. The tasks of the firefighters mainly involve rescuing people, animals, or property. CSI police, on the other hand, need to collect and provide evidence that may lead to conviction. Finally, the impact of their work as well as the likelihood of rewarding is different. Firefighters' actions have a salient meaning and receive immediate feedback as it usually determines whether the event would be defined as successful or not. CSI actions have little or no effect neither on the event or its outcome. Furthermore, it may take much longer before their actions would be rewarded. Studies have shown that these distinctions may affect not only the way first responders perceive themselves and their occupation but also their tendency to develop assorted psychopathologies including PTSD (Brough, 2004; Basiñska and Wiciak, 2003; for review see Langan-Fox and Cooper, 2011).

These differences may also affect the way firefighters and CSI police perceive and scan the environment during emergency events. While firefighters, who need to quickly respond and neutralize possible dangers, may have a more global perception and look at the general context (e.g., locate trapped people or scan for possible hazards such as gas balloons), CSI police are more focused on the small details, looking for evidence that may help solving the crime (e.g., traces of people who set up the fire or a hair of a murderer). Interestingly, these differentiations may suggest that people who are trained to do specific tasks may become more vulnerable in that very same aspect. A more global perception may improve context awareness, and contribute to the successful function of firefighters, but it may also result in an impaired ability to reverse negative contextual associations. In a similar way, focusing on details and detecting possible cues in the scene are essential skills for CSI police, but it may also lead to impairments in reversing the outcome of negative cues. Alternatively, it is possible that the different impairments are not due to different practice but a result of job selection. Hence, individuals who are more aware and capable to respond to stimuli in the general environment may have greater chances to become firefighters. While people with exceptional attention to detail, are more likely to become CSI police.

These possible explanations can be tested in several ways: first, a longitudinal study of new recruits can test cue and context reversal learning before traumatic exposure and compare it to performance at different stages of active service. Second, a study of active duty first responders may test interactions between cue and context reversal learning and tendency toward global or analytic perception. Finally, a third study may aim to test the effect of global and analytic practice on cue and context reversal learning in trauma exposed and unexposed students. These studies may help to distinguish between the effects of repeated traumatic exposure, job requirement and training and job selection and shed light on the possible interactions between these factors.

One may claim that the differences in the performance between the two groups relate solely to job selection and different training. Hence, since firefighters and CSI police are prone or trained to attend to specific stimuli in the environment, they experience difficulties in these domains, similar to a taller person who might be a great basketball player, but finds it difficult to enter narrow and short places. However, if this were the case, we would expect to find impairments not only in conditions of negative to positive reversal learning but also in conditions of positive to negative reversal learning. The fact that the observed impairment was selective to negative conditions, together with our previous findings showing deficits in the same direction in PTSD individuals (Levy-Gigi et al., 2014) suggest that these impairments are specific to trauma-exposed individuals.

Another way to revoke this potential claim is by comparing firefighters and CSI police to unexposed matched controls with similar training. For example, compare CSI police to civilians who work on a production line in an industrial factory and trained to pay close attention to detail. Similarly, compare firefighters who work in fire and rescue stations across the country to firefighters who work in airports and have similar training but only low (if any) level of traumatic exposure. These comparisons would allow controlling for possible effects other than traumatic exposure. It should be noted that our unexposed sample consisted of civilians who work at different tasks in an industrial factory, some require attention to context while others require attention to details, and yet there were no differences in their performance as a function of the specific job they fulfill.

Finally, it can be claimed that the definition of cue and context in our paradigm is only a semantic distinction and participants actually perceive the presented object and its background color as two different types of cue. In order to understand this claim it should be noted that the distinction between cue and context can be established through bottom-up or top-down processing. By bottom-up processing, we mean that the relationship is established trough gestalt rules according to the nature of the stimuli (Rock, 1975; Smith et al., 1978; Peterson, 1994). In top-down processing the stimuli are equivalent (e.g., pairs of faces, words or objects), and the cue-context relationship is usually established through explicit instructions on how to process them (e.g., Baddeley and Woodhead, 1982; Reder et al., 2002). Background color is a common example of context manipulation through bottom-up processing. Importantly, this manipulation produces similar effects as environmental manipulations (e.g., Macken, 2002; Rutherford, 2004; Isarida and Isarin, 2007; Hockley, 2008; Lang et al., 2009; van Ast et al., 2013). Therefore, it is very likely that the background color serves as context while the object serves as cue in our paradigm. However, in order to provide further validation for this cue-context relationship, future studies may aim to replicate our results while using top-down processing in a counter manner. Hence, explicitly instructing participants to focus on the background color instead of the object. If it indeed serves as context in the present study, such counter-instructions will result in impaired color- reversal in CSI police and impaired object reversal in firefighters.

Similar to our previous findings we showed no significant associations between performance on the cue-context reversal paradigm and PTSD levels, or any of the self-report measures. These results emphasize the hidden nature that may characterize the price of repeated traumatic exposure, suggesting that possible effects of traumatic exposure are not sufficiently defined by the standard measures of PTSD symptoms and diagnosis. Future cross-sectional studies may aim to test a larger sample of highly exposed individuals from various occupations in order to test the relationship between different symptoms and cue-context reversal learning as well as to further understand the impact of this hidden price on different aspects of life.

In conclusion, the present study provides further evidence for a possible hidden price of traumatic exposure, which is independent of PTSD symptoms and diagnosis as defined by the standard existing measures. In addition, it reveals that first responders from different occupations display diverse deficits and hence may pay different price. Future studies may aim to further examine the neural and behavioral nature of this hidden price and its impact on other aspects of daily life in various populations of first responders.

## Author contributions

Einat Levy-Gigi- design of the paradigm, design of the study, responsible for recruiting and testing participants, analyzing data, writing and finalizing the manuscript; Gal Richter-Levin- design of the study, writing and finalizing the manuscript; Szabolcs Kéri- design of the paradigm, analyzing data; writing and finalizing the manuscript.

### Conflict of interest statement

The authors declare that the research was conducted in the absence of any commercial or financial relationships that could be construed as a potential conflict of interest.
